# Validation of the WHO-5 Well-Being Scale among Adolescents in Ghana: Evidence-Based Assessment of the Internal and External Structure of the Measure

**DOI:** 10.3390/children9070991

**Published:** 2022-07-01

**Authors:** Frank Quansah, John Elvis Hagan, Francis Ankomah, Edmond Kwesi Agormedah, Regina Mawusi Nugba, Medina Srem-Sai, Thomas Schack

**Affiliations:** 1 Department of Educational Foundations, University of Education, Winneba P.O. Box 25, Ghana; fquansah@uew.edu.gh; 2 Department of Health, Physical Education and Recreation, University of Cape Coast, Cape Coast 03321, Ghana; 3 Neurocognition and Action-Biomechanics-Research Group, Faculty of Psychology and Sports Science, Bielefeld University, Postfach 10 01 31, 33501 Bielefeld, Germany; thomas.schack@uni-bielefeld.de; 4 Department of Education and Psychology, University of Cape Coast, Cape Coast 03321, Ghana; francisankomah57@gmail.com (F.A.); regina.nugba@ucc.edu.gh (R.M.N.); 5 Department of Education, SDA College of Education, Asokore-Koforidua P.O. Box AS 18, Ghana; 6 Department of Business & Social Sciences Education, University of Cape Coast, Cape Coast 03321, Ghana; edmond.agormedah@ucc.edu.gh; 7 Department of Health, Physical Education, Recreation and Sports, University of Education, Winneba P.O. Box 25, Ghana; mssai@uew.edu.gh

**Keywords:** adolescents, depression, distress, Ghana, health, secondary school, well-being

## Abstract

The WHO-5 well-being measure happens to be one of the most renowned measures of subjective well-being across the globe. Although the instrument has been calibrated in different countries, its psychometric properties and applicability in Africa, especially in Ghana, are not known. In this study, the WHO-5 well-being scale was validated among adolescents in Ghana by assessing the validity evidence of the measure based on the internal and external structure. In particular, the study examined the (1) dimensionality of the WHO-5 well-being scale, (2) quality of the items (including the scale functioning) for the measure, and (3) criterion validity of the well-being measure. Using a survey approach, 997 adolescents were recruited in secondary schools across the northern belt of Ghana. The study found a one-factor structure of the scale, which supports the factor solution of the original measure. The items were found to be of high quality, except for one item. The WHO-5 well-being measure was found to have sufficient evidence regarding convergent and divergent validity. The outcome of this validation study provides support for the validity and reliability of the WHO-5 well-being scale’s utility and use among adolescents in Ghana. The study encourages further validation studies to be conducted in Ghana to widen the reproducibility of the WHO-5 well-being measure.

## 1. Introduction

Adolescence is a critical stage of life characterised by rapid biological, emotional, and social development. In the context of positive psychology and mental health promotion, adolescents’ subjective well-being (SWB) is a central construct that is primarily used to examine one’s perceived quality of life [1]. Adolescents’ SWB is a complex construct that concerns optimal psychological functioning and experiences. It encompasses negative aspects such as the presence of depression and anxiety as well as positive facets such as contentment, satisfaction, and happiness [2]. The concept also represents a vital resource for positive development and a prerequisite for successful learning [3,4]. Adolescents, per their stage of development, are vulnerable to intersecting mental health stressors and vulnerabilities. The emergence of the COVID-19 pandemic heightened mental health issues (e.g., anxiety, depression) across the adolescent population, and this was further exacerbated by financial distress and/or poverty, including other structural disparities [5,6,7]. Recent studies, for example, have established that the COVID-19 crisis affected adolescents’ well-being and positive development, which resulted in a greater risk of anxiety and depression [8,9,10].

Although several instruments exist for assessing the psychological or subjective well-being of individuals, the World Health Organization Well-being Index (WHO-5), happens to be one of the prominent and widely used inventories for measuring SWB [11,12]. The development of the scale began with its longer versions, the WHO-28 and WHO-10 [13]. By 1998, researchers had successfully reduced the instrument to a more user-friendly five-item scale with a six-point Likert scale, ranging from zero (at no time) to five (all of the time) [14]. Since its first publication in 1998, the WHO-5 has been translated into more than 30 languages and has been used in research studies all over the world. In addition, the scale (WHO-5) has been applied in different fields/disciplines using different population groups with diverse health conditions including patients with cancer [15,16]; Type 1 and 2 diabetes [17,18,19,20]; depression [21,22]; suicidal behaviors [23,24]; cardiovascular disease (e.g., myocardial infarction patients) [25,26]; alcoholism and other substance use disorders [27,28,29]; geriatrics [30]; stroke [31]; sleep disturbances [32]; personality disorder [33]; grief [34]; and occupational psychology [35]. It has also been applied to adolescents and students [36,37,38].

Numerous studies have assessed the validity and psychometric properties of the WHO-5 well-being index across different geographical contexts such as in Europe [35,39,40], Asia [41,42,43,44], and Africa [45,46]. For example, in Europe, Sischka et al. [35] used item response theory and the analysis of measurement invariance across 35 countries and discovered that the model appropriately fitted the data for all countries. The WHO-5 showed an overall high level of reliability. Measurement invariance analyses revealed metric invariance but discovered scalar invariance across countries. Using item response theory, Lucas-Carrasco et al. [47] in Spain confirmed that the total score of the WHO-5 has a sufficient statistic, and an adequate level of sensitivity (61%) and specificity (84%). Cichoń et al. [39], using Polish adults with diabetes, discovered one-factor structure (unidimensional factor structure) of the Polish version of the WHO-5. The internal consistency and discriminant validity indices of the scale were satisfactory. Similar findings have been reported in Turkey [40]; Denmark [12,48]; Spain [49]; Sweden [50,51]; the Netherlands [11,52]; Germany [53,54]; Switzerland [21]; and Australia [17]. In Asia, Fung et al. [43] in China found that the WHO-5 is unidimensional with good internal consistency and concurrent validity, and a good model fit. Furthermore, Faruk et al. [42] in Bangla found a one-factor structure for the scale with the exploratory factor analysis (EFA) explaining more than one-third of the total variance. Similar findings were also reported in other studies in China [55,56]; Japan [23,57,58]; Iran/Persia [41,44,59,60]; Thailand [61]; and Lebanon [62]. In Africa, Seb-Akahomen et al. [46] established that the WHO-5 well-being index has satisfactory validity as a screening tool for the detection of depression in Nigeria. The sensitivity and specificity values obtained were 0.857 and 0.851, respectively. Moreover, Chongwo et al. [45] in Kenya established one-factor structure of the Swahili version of the WHO-5 well-being index. The scale demonstrated good psychometric properties in terms of internal consistency and discriminant validity.

Given the growing evidence of worsening mental health and increased levels of distress, emotional isolation, depression, and anxiety amongst adolescents and young people, against a backdrop of exacerbated psycho-social risk factors such as bereavements, disruptions in education and life routines, and poor parental support and control [63,64,65], an accurate measure of well-being is required taking into consideration cultural disparities. Essentially, the concept of well-being is context- and cultural-driven and consequently differs based on geographical situations [66,67]. Supporting these variations, Bull et al. [68] established that adolescents in the Northern region are happy when they have food in stock. WHO-5 may yield varying applicability within and across other contexts (e.g., Africa), often noted with a collectivist identity of its setting, as opposed to the individualistic background of most Western countries. Indeed, individualistic identity and collectivist culture influence the measurement of well-being. For example, people from a collectivistic culture tend to emphasize interpersonal social affect, whereas those with an individualistic background focus on intrapersonal positive affect. Consequently, it has been shown that people from an individualistic culture will usually provide higher ratings as compared to those from a collectivist culture, which can lead to varying levels of functioning of the items and the measure in general [69,70].

The applicability of the WHO-5 in other geographical boundaries, such as Ghana, is not known and may not be well understood. Although there have been few validation studies in Africa, their findings are not applicable to the Ghanaian context for two reasons. First, research has shown that the conceptualization of well-being in Ghana varies from other African countries, with the variations being large in non-West African nations [71]. According to Osei-Tutu et al. [71], these cultural variations manifest in how the concept of well-being is conceptualised in these nations and consequently, how the well-being variable is measured. Secondly, this study has a unique sample in terms of their vulnerability and age group. Previous validation studies used samples that suffered from some medical conditions (e.g., HIV patients) or have been diagnosed with a psychological condition. In contrast, the sample for this study had not been diagnosed with any medical or psychological conditions but was believed to have been in a deprived situation. These sample characteristics could affect the measurement and conceptualisation of the well-being measure.

To date, the psychometric properties of the WHO-5 have not been tested in many West African countries, with none documented in Ghana, despite its wide usage in the country [72,73]. Adapting and validating the WHO-5 in Ghana provides useful information to guide the utility of the scale for assessing SWB among adolescents. Besides, context-specific differences suggest that further studies are warranted to ascertain the applicability of the WHO-5 using different samples with a modern non-sample dependent measurement procedure such as the item response theory (IRT). The purpose of the study was to validate the WHO-5 well-being scale among adolescents in Ghana. In particular, the study examined the (1) dimensionality of the WHO-5 well-being scale, (2) quality of the items (including the scale functioning) for the measure using unidimensional graded response model, and (3) criterion validity of the WHO-5 well-being measure. In addition, the measurement of affect using the International Positive and Negative Affect Schedule, Short Form (I-PANAS-SF) in this study provides an understanding of the convergent and divergent validity evidence of the WHO-5 index.

## 2. Methods and Materials

### 2.1. Study Setting

The study was carried out using adolescents in senior high schools (SHS, also referred to as secondary school) in the northern belt of Ghana. The focus was on this setting due to the unique characteristics of the regions within the northern zone of the country. According to Ofori-Boateng and Bab [74], over 40% of the populace in the northern zone are living in poverty, with the Upper West and Upper East being the most affected. For example, 9 out of 10 and 8 out of 10 people in the Upper East and Upper West regions, respectively, live below the poverty line, based on the Ghana poverty reduction strategy paper. Further, basic school completion rates are very low in the northern zone [74,75]. In a recent report by the World Bank [76], poverty was found to be increasing in the northern zone (e.g., Upper West, Savannah, and Upper East), with the majority of its settlements engaged in subsistence agricultural farming. The World Bank [76] report further indicated that the regions in northern Ghana, had fewer chances of breaking out of poverty, irrespective of the employment sector. These peculiarities identified in this setting served as the motivation behind the choice of the zone for this research. The reason is that the increasing rate of poverty in Ghana has higher chance of affecting the well-being of these individuals, especially adolescents who expectedly depend solely on their poor parents [77,78,79].

### 2.2. Sample Characteristics

Approximately an 87 percent response rate (*n =* 997) was obtained with an initial sample expectation of 1200 adolescents in secondary schools in two randomly selected regions in the northern zone of Ghana (i.e., Upper West Region and Savannah Region). The excess of 203 respondents represents those who either did not provide any response (or little response) or opted out of the study. A larger proportion of the adolescents were males (*n =* 496, 49.7%), 47.7% (*n =* 476) were females, and 2.5% (*n =* 25) were identified as diverse. The youngest participant was 14 years, whereas the oldest was 21 years old. The mean age was 19 years with a standard deviation of 1.5. The majority of the participants were in their 2nd year (*n =* 668, 67%) whereas 33% (*n =* 329) were in their 1st year. The third years had written their final examination and were no longer in school at the time of data collection. The study employed the multi-staged sampling technique. First, a simple random sampling was adopted to sample two regions in northern Ghana. In each region, five schools were selected based on cluster sampling. Afterwards, the purposive sampling technique was then used to roll in the participants for the study. The following were the eligibility criteria for the study: (1) participants who have stayed and schooled within any of the five northern regions for over 10 years, (2) willingness of participants or their guardian to provide consent/assent, (3) not necessarily being an indigene of any of the five northern regions, and (4) being able to read and write in the English language.

### 2.3. Measurement of Variables

#### 2.3.1. Word Health Organization 5-Item Well-Being Index

The WHO-5 well-being index is a brief measure of well-being for a 2-week period. The short-scale was developed by the WHO [14] (see https://www.psykiatri-regionh.dk/who-5/Documents/WHO-5%20questionaire%20-%20English.pdf, accessed on 17 February 2022). The scale is unidimensional with 5-items measuring well-being on a 6-point scale (0—at no time, 1—some of the time, 2—less than half of the time, 3—more than half of the time, 4—most of the time, 5—all of the time). The items include: “I have felt cheerful and in good spirit”, “I have felt calm and relaxed”, “I have felt active and vigorous”, “I woke up feeling fresh and rested”, and “My daily life has been filled with things that interest me”. The instrument has gained popular usage and its psychometric properties have been established across different cultures and with a diverse sample. In this study, a reliability estimate of 0.754 was reported using of Omega ω estimation procedure.

#### 2.3.2. International Positive and Negative Affect Schedule, Short Form

The I-PANAS-SF [80] is a short version of the original 20-item PANAS instrument [81]. The I-PANAS-SF is a 10-item two-mood scale which measures positive affect (PA) and negative affect (NA). Affect is defined as a psychological trait that denotes mental states embracing evaluative feelings [82]. The PA domain is characterized by feelings of pleasurable engagement with a high level of energy, alertness, and enthusiasm. The NA, on the other hand, is characterized by emotionally distressing states, and feelings of sadness, fear, and unfriendliness [81]. The items for the PA domain were determined, attentive, alert, inspired, and active. The NA dimension had items which included afraid, nervous, upset, ashamed, and hostile. Respondents were required to indicate the extent to which they experienced each of the listed emotions within the last 2 weeks, using a 5-point scale option: very slightly or not at all—1, a little—2, moderately—3, quite a bit—4, very much—5. Several scholars have found the PANAS as an accurate measure of emotions [83,84,85]. The reliability estimate for this scale (in this study) using the Omega ω procedure was 0.819.

### 2.4. Ethical Concerns and Quality Control

The data were obtained based on the ethical approval from the Institutional Research Board (IRB) of the University of Education, Winneba, with reference number, DAA/P.1/Vol.1/39. The approval date was 10 March 2022. The headteachers and the regional education offices of the various selected schools were contacted and official letters were sent to them for approval of the data collection. After all approvals, we recruited and trained 10 research assistants who were indigenes of the two selected regions. These assistants had backgrounds in public health, health education, psychology, and measurement and evaluation, with a first degree as the minimum qualification and a Master of Philosophy degree as the highest educational qualification. All of the assistants had prior experience in data collection of this form. The assistants were trained on the purpose of the study and how to administer the WHO-5 scale. During this training, the items and response options were carefully discussed, following the approaches of previous validation studies [86,87]. Although there was no translation of the scale, the research assistants were permitted to explain some of the items in the local dialects when necessary. Before the administration phase, consent forms were signed by (a) the participants who were 18 years and above and (b) parents who had wards below the age of 18 years. The purpose of the study was well explained to the participants. All ethical considerations such as confidentiality, anonymity, protection of vulnerable participants, protection from harm, and volition, among others, were adhered to. The data collection period lasted 3 months and the duration for completion was between 15–20 min for each participant.

### 2.5. Statistical Analyses

The sample size for item response theory analysis has been a subject of contention in the relevant literature [88]. However, a greater consensus for simulation studies has resolved that a minimum sample of 500 should be enough for the accurate estimation of parameters [89]. This research used 997 cases, which falls within the recommended sample size. First, EFA was carried out with the FACTOR computer programing software (version 12.1, Rovira i Virgili University, Tarragona, Spain) to establish the dimensionality of the WHO-5 well-being scale. Parallel analysis based on the minimum rank factor was used as the factor extraction method, grounded on the recommendations of Timmerman and Lorenzo-Seva [90]. The Promin rotation approach was also utilized in the EFA due to its superiority, as reported in the literature [91]. The EFA was conducted using the bootstrapping method with 5000 bootstrap samples.

Further, a graded response model was specified with a single dimension to test the quality of the items and their respective contributions to the measurement of the construct of interest (i.e., well-being). The IRT approach provides an accurate measurement that caters for the varying levels of ability in responding (in relation to their SWB) in the subject population, which earlier studies have not established. The IRT PRO computer programme (Version 4.2.21711.3002, Scientific Software International Inc., Skokie, IL, USA) was used to process the data [92] for the graded response analyses. The slope parameter was interpreted based on Reckase and McKinley’s [93] criteria that values > 1.0 to 1.5 reflect good items, but estimates above 1.5 show high-quality items. For the difficulty indices, a threshold step greater than 0.80 was required [94]. Trace lines, information curves, and test characteristic functions were also used for interpreting the results.

As an assumption, the percentage of responses falling within each of the 6 scale categories was explored. It was found that very few of the category options had percentage cases below 10%, with the majority of them having responses over 10% (see Table 1). This shows that there were enough cases within each response option for an item response analysis to be conducted [95]. The local dependency hypothesis was tested to have an idea of whether the responses provided for each item are due to the construct measured or some other factors such as language difficulty. The outcome of the local dependency test yielded values between 2.0 to 4.8 which were considered small and thus, had a little influence on model fit indicators [96].

Validity evidence based on the external structure of the measure was also assessed through correlation matrix and multiple regression analysis using SPSS (version 25, International Business Machines (IBM) Incorporation, New York, NY, USA). This was carried out by correlating and regressing the WHO-5 well-being index with positive and negative affect measures (using the I-PANAS-SF).

## 3. Results

### 3.1. Dimensionality of WHO-5

#### 3.1.1. Model Fit

The Kaiser-Meyer-Olkin (KMO) test yielded a value of 0.819 with a confidence interval of 0.796 and 0.831. The measure of sampling adequacy showed values between 0.817 and 0.820. Generally, the goodness of fit indicators showed good model fit for the 5-item unidimensional model which was fitted. For example, values of the Root Mean Square of Residuals [RMSR = 0.0572, CI (0.038, 0.070)] and Weighted Root Mean Square Residual [WRMSR = 0.0567, CI (0.039, 0.069)] provided sound support for the fit of the specified model.

#### 3.1.2. Parallel Analysis

The results in Table 2 revealed one-factor solution for the WHO-5 well-being scale. The single factor structure accounted for about 88.44% of the variability in the measure of well-being. The output of the parallel analysis based on the minimum rank factor is shown in Table 2.

#### 3.1.3. Item Quality Based on Slope, Difficulty Thresholds, and Reliability

The item parameters for the WHO-5 well-being items are presented in Table 3.

As shown in Table 3, all the items were of high quality (with a slope parameter greater than 1.5), with the exception of WB1 “I have felt cheerful and in good spirit” which had a discrimination parameter below 1.5. Although the slope value for WB1 was good, it was not of high quality as compared to the rest of the 4 items. Further, the difficulty threshold appeared to increase monotonically, which is a characteristic of polytomous item response models. However, some of the threshold steps were not functioning as intended. Most notably, the threshold steps among *b*_3_, *b*_4_, and *b*_5_ were very small and did not meet the recommended 0.81 threshold [93]. For example, the threshold step between *b*_3_ (Less than half of the time vs. More than half of the time) and *b*_4_ (More than half of the time vs. Most of the time) were 0.54, 0.25, 0, 0.15, and 0.21 for WB1, WB2, WB3, WB4, and WB5, respectively. A similar trend of results was found for the threshold step between *b*_4_ (More than half of the time vs. Most of the time) and *b*_5_ (Most of the time vs. All the time).

**Figure 1:**
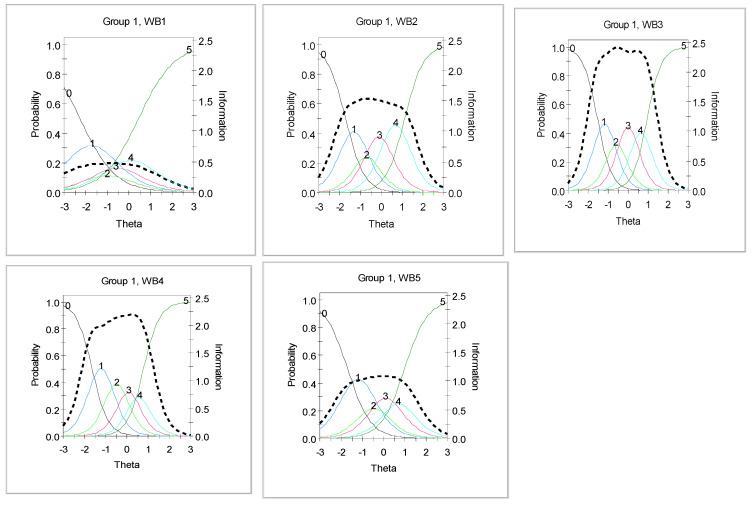
Trace lines and information function curves for WB1, WB2, WB3, WB4, and WB5.

The trace lines and information curves for the items are further illustrated in Figure 1.

Observing the results from Figure 1, it is clear that each of the items offered unique empirical information to the measure of well-being, with WB1 (‘I have felt cheerful and in good spirit’) providing the least information and WB3 (‘I have felt active and vigorous’) provided the largest empirical information. No item was found to be redundant in the measure of well-being (see Figure 1). It can also be observed that some of the scale options did not function as intended for the items. For example, options 2, 3 and 4 were not efficiently utilized for item 1. Similar instances were found across other items with options 3 and 4 being predominant.

**Figure 2:**
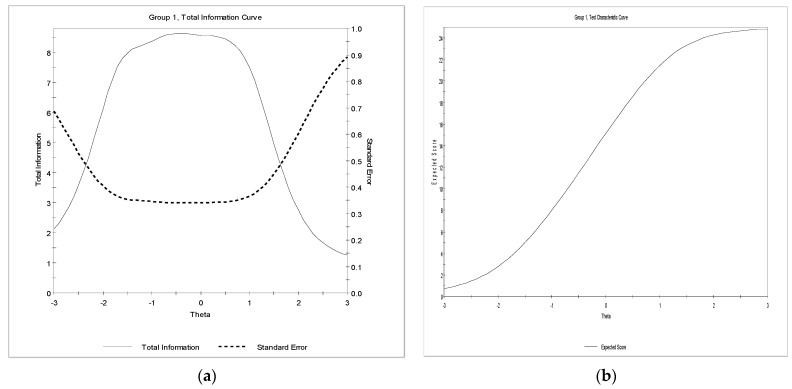
(**a**) Total information function (**b**) Test characteristic curve.

The total information curve, as shown in Figure 2a, showed that the function increased with decreasing standard error, at least for a particular area covered. The reliability estimate of 0.86 was revealed, indicating some sufficient degree of precision for the area covered by the items and consequently, how the WHO-5 well-being scale works [97]. Most importantly, the function provides relatively stable information between −1.2 to 0.80, with maximum information provided at an ability level of −0.40. The test characteristic curve also supports the degree of precision in terms of the association between ability and expected scores (see Figure 2b). Thus, increasing levels of ability were found to be associated with increasing true scores.

### 3.2. Criterion Validity

We also examined validity evidence based on external structure through criterion validity by assessing how the WHO-5 well-being index is associated with positive and negative affect measures. The details of the analysis are presented in Table 4.

The outcome of the analysis revealed a positive correlation between PA and WHO-5 well-being measure (*r =* 0.576), and a negative correlation between NA and WHO-5 well-being measure (*r =* −0.676) (see Table 4). Additional results showed that both PA (β = 0.132, *t =* 4.670, *p <* 0.001) and NA (β = −0.657, *t =* −23.156, *p <* 0.001) significantly predicted well-being. Notably, PA and NA explained about 56.6% of the variances in the SWB of adolescents.

## 4. Discussion

This study validated the WHO-5 well-being scale among adolescents in Ghana, specifically, by examining the dimensionality, the quality of the items, and how the measure correlates with the feeling of affection. The internal structure of the WHO-5 well-being measure was validated through the application of the unidimensional graded response model of the item response theory. The outcome of the EFA points to a unidimensional scale which accounted for close to 90 percent of the variability in well-being. The scale also showed high internal consistency [98,99], and high factor determinacy, indicating a strong correlation between the estimated ability (well-being) and the true ability. These results are indications that the one-factor structure estimates better regarding the well-being of adolescents. These findings are in agreement with previous validation studies [14,20,39,42,43,46,48]. This implies that the well-being of adolescents in Ghana could be explained by a single underlying trait likewise adolescents of other countries. This supports the applicability of the WHO-5 scale within the Ghanaian setting as a unidimensional measure of well-being. Additionally, it can be said that whether clinical population or the general population, a single latent trait appears to better explain the well-being of adolescents.

Except for item WB1 (I have felt cheerful and in good spirit) which was good, all the items had high discrimination indices suggesting excellent functioning. The implication is that the item had relatively low discrimination and could not distinguish excellently between the levels of well-being as compared to the other four items [93]. We, therefore, argue that, among the adolescents in Northern Ghana, feeling cheerful and being in good spirit might not necessarily bring about high or low well-being. Bull et al. [70] reported in their study that adolescents in the Northern zone of Ghana are extremely happy when food was in stock. Such instances would probably make these adolescents value food over other indicators of well-being. Further investigation could examine the utility of this particular item as a proxy for measuring well-being. This item also appears to be double-barreled, in the sense that ‘feeling cheerful’ and ‘being in good spirit’ may describe different states of well-being at a particular point in time. Hence, the respondents are more likely to be confused as to whether they are responding to the state of ‘feeling cheerful’ or ‘being in good spirit’. Although the different aspects of the statements have similar meanings, it can be said that one can appear cheerful, but might be in a bad spirit. This is not surprising because instinctively, individuals in bad states (i.e., covert) still want to outwardly look (i.e., overt) cheerful before others would not notice what they might be going through. Cultural reasons may help explain the current observation. In many African societies, individuals are overtly expected to show bravery, resilience, and show hardiness toward unpleasant life experiences that may generate psychological episodes such as anxiety, fear and distress. Individuals who overtly display these unpleasant emotional reactions are touted as weak and lack resilience. Such people are often confronted with shame and potential rejection because of their perceived cowardice attitudes amid challenging situations [100].

In terms of the response categories, two items (WB2 and WB3) had all the response categories not functioning efficiently. However, items WB1, WB4, and WB5 saw response categories b_3_, b_4_, and b_5_ as problematic [94]. Generally, the problematic functioning of the response categories implies that response options such as 3—‘more than half of the time’, 4—‘most of the time’, and 5—‘all of the time’ were not well understood by the respondents. Inferring from the reference point of the measure of well-being in this study, the adolescents were to indicate how they have been feeling for the past two weeks using the aforementioned response options. Of particular concern is the use of the word ‘time’ across all the response options. Does the word ‘time’ represent every other day within the two weeks or some time periods in a day for the two weeks because well-being was measured as a state and not as a trait in this study. For instance, if a respondent chooses the response option ‘more than half of the time’ for an item ‘My daily life has been filled with things that interest me’, could this mean the respondent’s life has been filled with things that interest them for more than 14 days? Or collectively, more than the number of times within each day across the 14 days they have felt they have been filled with things that interest them? If interpretations from both angles hold, then the use of the word ‘time’ could amount to the reason for non-clarity among the response options. Further studies are required to explore the functioning and use of the response options.

In totality, an increasing level of well-being was correspondingly associated with the true score. There was high precision with the information provided by items on the well-being scale, and this was particularly stable for abilities ranging from −1.2 to 0.80 (slightly low to slightly high). The peak of the information was at an ability level of −0.40. This ability level is close to 0 (normal well-being), suggesting that among the Ghanaian adolescent population, the WHO-5 well-being scale provides enough information on those who are slightly low to slightly high in terms of well-being. Despite the few issues with the response functioning, the well-being scale has shown high-quality items which may serve as good proxies for the measurement of well-being among the adolescent population in Ghana.

The results of this research corroborate with previous studies conducted in countries which have a similar cultural identity as Ghana. For example, the WHO-5 validation study by Khosravi et al. [59] which was conducted in a collectivist culture (i.e., Iran) such as Ghana revealed a similar trend of results. Specifically, Khosravi and co-authors found the first item of the WHO-5 (“I have felt cheerful and in good spirit”) contributed the least to the measure of well-being. On the contrary, Bonsignore et al.’s [54] validation study showed very high rates of sensitivity in detecting depression among Germans, with an individualistic background. This confirms the observations of scholars that people from the individualistic culture are more likely to have high ratings on WHO-5 as compared to those with collectivist backgrounds [69,70].

The results further showed sufficient validity evidence based on the external structure of the measure. The WHO-5 well-being measure was found to be positively related to PA and negatively associated with NA. This relationship has a grounding in literature that high levels of PA are associated with lower levels of psychological distress and improved well-being and vice versa, whereas a high level of NA was found to be related to deteriorating well-being [101,102]. Corroborating these findings, other validation studies confirmed that feelings of affect significantly predicted depression and well-being [83,84]. This finding supports the sufficiency of the evidence of convergence and divergence of the WHO-5 well-being measure.

### 4.1. Limitations and Future Studies

Despite the contribution of these research findings to literature and practice, there were some limitations. First, the sample of adolescents used was those in-school at the time of the study. This presupposes that other adolescents within the study area who are either not schooling or have dropped out of school were not sampled. Consequently, the generalization of these findings might be limited to those adolescents in schools. There is a need for such groups of individuals to be included in further studies. Secondly, even though the WHO-5 well-being index has been found to detect some level of depression among clinical samples, this has not been tested within the Ghanaian context. We encourage future researchers to validate this instrument with clinical samples and as well evaluate the Minimum Clinical Important Difference (MCID) of this measure.

### 4.2. Practical and Clinical Implications

The outcome of this research endorses the utilization of the WHO-5 well-being measure among adolescents in Ghana. The study contributes meaningfully to the psychometric literature regarding the validity and reliability properties of the WHO-5 index. Hence, other scholars are encouraged to use this measure for screening psychological distress symptoms in their studies. Moreover, researchers who wish to test the efficacy of some well-being-related interventions can adopt WHO-5 well-being due to its high evidence of validity. The findings provide enough grounds for clinical, school and health psychologists as well as school counsellors to use as a preliminary investigative tool for assessing depressive symptoms among adolescents.

## 5. Conclusions

The outcome of this validation provides support for the validity and reliability of the WHO-5 well-being scale’s utility and use among adolescents in Ghana. The one-factor structure of the scale was confirmed and the items were found to be of high quality. However, the scale options appeared problematic for use among the sample. The study encourages further validation studies to be conducted in Ghana to widen the reproducibility of the WHO-5 well-being measure.

## Figures and Tables

**Table 1:** Percentage of cases falling within each response category.

No.	Items	0	1	2	3	4	5
WB1	I have felt cheerful and in good spirit	9.7	14.3	7.0	11.6	17.3	40.0
WB2	I have felt calm and relaxed	9.5	14.5	11.3	22.9	24.1	17.7
WB3	I have felt active and vigorous	10.2	14.2	13.3	23.6	18.5	20.2
WB4	I woke up feeling fresh and rested	9.0	15.0	18.0	18.1	14.6	25.3
WB5	My daily life has been filled with things that interest me	11.4	19.7	13.1	19.1	13.9	22.8

0—at no time, 1—some of the time, 2—less than half of the time, 3—more than half of the time, 4—most of the time, 5—all of the time.

**Table 2:** Parallel Analysis.

Variable	Real-Data % of Variance	Mean of Random % of Variance	95 Percentile of Random % of Variance
1	72.6446 *	41.0347	57.2639
2	13.8067	29.9054	37.6526
3	10.4180	19.5669	26.9200
4	3.1307	9.4930	18.8668

* Advised number of dimensions: 1; Total Variance explained = 88.44%; Reliability = 0.850; Factor Determinacy Index = 0.922.

**Table 3:** Graded Model Item Parameter Estimates, logit: *a*(*θ*-*b*).

Label	*a*	*s.e._a_*	*b*_1_	*s.e._b_*_1_	*b*_2_	*s.e._b2_*	*b*_3_	*s.e._b_*_3_	*b*_4_	*s.e._b4_*	*b*_5_	*s.e._b_*_5_
WB1	1.22	0.09	−2.27	0.16	−1.19	0.10	−0.82	0.08	−0.28	0.07	0.44	0.07
WB2	2.21	0.13	−1.71	0.09	−0.92	0.06	−0.48	0.05	0.23	0.05	1.13	0.07
WB3	2.80	0.18	−1.53	0.08	−0.82	0.05	−0.34	0.05	0.34	0.05	0.95	0.06
WB4	2.64	0.17	−1.64	0.08	−0.80	0.06	−0.18	0.05	0.33	0.05	0.78	0.05
WB5	1.84	0.11	−1.69	0.10	−0.70	0.06	−0.22	0.05	0.43	0.05	0.98	0.07

*a*—discrimination parameter; *b*—difficulty threshold; Marginal Reliability for Response Pattern Scores: 0.86.

**Table 4:** Relationship between WHO-5 Well-being Measure and I-PANAS-SF Measure.

Variables	Positive Affect (PA)	Negative Affect (NA)
Positive Affect	1	
Negative Affect	−0.676 **	1
WHO-5 Well-being Index	0.576 **	−0.746 **
Beta	0.132	−0.657
*t*-value	4.670	−23.156
*p*-value	<0.001	<0.001

R^2^ = 0.566; Criterion variable for the regression: Well-being, ** significant at *p <* 0.001.

## Data Availability

The data are available upon reasonable request through the corresponding author.
